# Progressive age-associated activation of JNK associates with conduction disruption in the aged atrium

**DOI:** 10.1016/j.mad.2015.05.001

**Published:** 2015-03

**Authors:** Sandra A. Jones, Matthew K. Lancaster

**Affiliations:** a School of Biological, Biomedical and Environmental Sciences, University of Hull, Kingston-upon-Hull, HU6 7RX, UK; b Faculty of Biological Sciences, University of Leeds, Leeds LS2 9JT, UK

**Keywords:** Atria, Connexin43, Gap junction, Conduction velocity, JNK

## Abstract

Connexin43 (Cx43) is critical for maintaining electrical conduction across atrial muscle. During progressive ageing atrial conduction slows associating with increasing susceptibility to arrhythmias. Changes in Cx43 protein expression, or its phosphorylation status, can instigate changes in the conduction of the cardiac action potential. This study investigated whether increased levels of activated c-jun *N*-terminal kinase (JNK) is responsible for the decline of Cx43 during ageing. Right atria from guinea pigs aged between 1 day and 38 months of age were examined. The area of the intercalated disc increased with age concurrent with a 75% decline in C43 protein expression. An age-dependent increase in activated-JNK correlated with a rise in phosphorylated Cx43, but also slowing of action potential conduction velocity across the atria from 0.38 ± 0.01 m/s at 1 month of age to 0.30 ± 0.01 m/s at 38 months. The JNK activator anisomycin increased activated JNK in myocytes and reduced Cx43 protein expression simulating ageing. The JNK inhibitor SP600125, was found to eradicate almost all trace of Cx43 protein. We conclude that *in vivo* activation of JNK increases with age leading to the loss of Cx43 protein resulting in impaired conduction and contributing to the increasing risk of atrial arrhythmias with advancing age.

## Highlights

• Guinea pig atria from multiple ages ranging from neonate to old age were compared.
• Action potential conduction velocity showed a significant reduction in advanced age.
• Connexin43 protein reduced dramatically in the right atria with increasing age.
• An age-dependent rise in activated-JNK correlated with a rise in phosphorylated Cx43.
• JNK signalling is a mediator of gap junctional remodelling with increased age.

## 1 Introduction

Approximately 20% of the population are over 65 years of age, and by 2050 this is predicted to increase to over a quarter of the Western population (Cracknell, 2010). Ageing is considered to be the highest risk factor for cardiac arrhythmias. Ageing associates with progressive remodelling of heart muscle and a progressive increase in the incidence of conduction abnormalities, uncoordinated contraction and diminished myocardial function. In advanced age this is particularly evident within the right atria and sinoatrial node with an age-associated increase in sick sinus syndrome and atrial fibrillation (Centurión et al., 2005; Jones et al., 2004; Roberts-Thomson et al., 2009).

Cardiac myocytes are linked at their intercalated discs. These essentially consist of ‘mechanical junctions’ to bond cells together, and ‘gap junctions’ composed from a family of proteins known as connexins (Cx) responsible for intercellular communication and propagation of the action potential (for a fuller review see Noorman et al., 2009). Cx43 is the most abundantly expressed isoform in heart muscle with additional isoforms such as Cx40 and Cx45 occurring at comparatively low levels. Thus, Cx43 predominately forms cardiac gap junctions and its expression is critical for maintaining cardiac conduction throughout the heart, with the notable exceptions being within the His-Purkinje conducting system, sinoatrial and atrioventricular nodes (Jones et al., 2004; Nikolski et al., 2003). Even moderate changes in gap junctional conductance directly correlate with changes in cardiac conductance velocity highlighting their key role in ensuring normal conduction in the heart (Dhillon et al., 2014). Depletion of Cx43 has been shown to increase the risk of arrhythmias, and a similar change during progressive ageing may contribute to the increasing risk of arrhythmia susceptibility in old age (Danik et al., 2004).

The activation of stress-associated signalling within the heart progressively increases during ageing with a number of intracellular pathways in cardiac myocytes potentially evoked such as protein kinase A, protein kinase C and the intracellular signalling family of mitogen activated protein kinase (MAPKs) including p38, p42/44 and c-jun *N*-terminal kinase (JNK) (Powers et al., 2004; Baines and Molkentin, 2005). These pathways have been implicated in the regulation of cardiac conduction during episodes of acute and chronic stress by phosphorylation of the Cx43 protein at the C-terminus (Polontchouk et al., 2002; Remo et al., 2011).

Phosphorylation of Cx43 is complex in terms of regulation and effect. Basal phosphorylation of Cx43 in the heart ensures proper gap junction assembly and targeting to the intercalated disc with de-phosphorylation in instances such as ischemia leading to gap junction disruption (Beardslee et al., 2000). Phosphorylation effects however are complex. Enhanced phosphorylation has also been shown to have the potential to alter channel gating reducing conductance, alter selectivity, and lead to down-regulation of Cx43 expression (for review see Solan and Lampe, 2009).

The stimulation of acutely cultured single cardiac myocytes and HL1 cells *in vitro* with acute stress has been demonstrated to provoke increased levels of activated-JNK (phosphorylated JNK isoform) with a reduction in both Cx43 mRNA and protein levels, (Petrich et al., 2002; Ogawa et al., 2004; Yan et al., 2013). In contrast though other studies also using acute stress simulation in culture to provoke activated-JNK have shown enhanced Cx43 expression (Shyu et al., 2004; Salameh et al., 2009). Thus, the manipulation of JNK and the ensuing effect on Cx43 protein expression within myocytes remains controversial.

Studies of transgenic mice *in vivo* using the cre-LoxP-mediated system to express MKK7D (a specific activator of JNK under the control of the murine α-MHC promoter) demonstrated that cardiac specific over-expression of activated-JNK resulted in a 90% reduction of Cx43 protein expression compared with their control littermates (Petrich et al., 2004). This was accompanied by the functional affect of a 40% reduction in action potential conduction velocity and sudden death of the transgenic mice at 6–8 weeks highlighting the potential arrhythmogenic consequences. Furthermore, using cardiac myocytes from MKK7D over-expressing mice the activity of JNK was inhibited *in vitro* and shown to attenuate Cx43 protein expression (Petrich et al., 2002). Thus, the transgenic over-expression mouse model illustrates JNK acts as an important mediator in the regulation of Cx43 expression. This is further supported by the recent finding that in rabbits activation of JNK with anisomycin lead to a 34% decline in Cx43 associating with a 50% increase in activated JNK within the left atria. This was accompanied by an increased susceptibility to atrial arrhythmias (Yan et al., 2013).

Cx43 protein has previously been identified as a molecular correlate inversely linked to susceptibility of atrial arrhythmias (Kostin et al., 2002). Atrial arrhythmias, in particular atrial fibrillation, show increasing prevalence with age and are present at a high incidence within the elderly population (Kostin et al., 2002; Brembilla-Perrot, 2003). Previous studies illustrate the relationship of the protein levels of activated-JNK to Cx43, along with Cx43 phosphorylation status, indicating their potential significance in the mechanistic underpinnings of a higher risk for atrial arrhythmias in old age. Therefore our hypothesis was that progressive activation of JNK signalling and subsequent modification of Cx43 contributes to the “aged-heart” phenotype; a phenotype predisposed to atrial arrhythmias.

## 2 Materials and methods

### 2.1 Animal model

Healthy female tricolor guinea pigs were obtained at 1 day (0.03 month), 1 month, 18 months, 26 months and 38 months of age (*n* = 5 per age group). This age range covers the previously determined life expectancy of this in-bred guinea pig strain (Jones et al., 2004). All animal procedures were performed in accordance with the United Kingdom Animals (scientific procedures) Act 1986 and reviewed by the University of Hull and Leeds Biomedical Sciences ethics committees. Animals were humanely sacrificed by intravenous administration of an overdose of pentobarbital followed by removal of the heart and isolation of the right atrium.

### 2.2 Extracellular electrode recording

The intact right atria was pinned endocardial surface uppermost in bicarbonate buffered Tyrode’s solution, maintained at 37 °C, and continued to generate spontaneous impulses (Jones et al., 2004). Using two extracellular modified bipolar electrodes, one as a stationary reference electrode and the second as a moving electrode, the local action potential activation time across the muscle was measured as previously described (Yamamoto et al., 1998). For each animal examined, action potential conduction was determined in the main direction of propagation in the atrial muscle – perpendicular to the crista terminalis.

### 2.3 Immunofluorescence

Single cardiac myocytes were isolated from right atrial tissue by enzymatic digestion (Lancaster et al., 2004). Cells or 10 μm frozen sections were subjected to immunolabelling as previously described (Jones et al., 2002). Details of the primary antibodies utilised are listed in Table 1. FITC-conjugated anti-rabbit or anti-mouse antibodies, as appropriate for the primary antibody (Dako, Denmark) were used to fluorescently tag the primary antibody. Following immunolabelling, myocytes were incubated for 2 h with wheat germ agglutinin (WGA) conjugated to rhodamine, a lectin that binds to *N*-acetylglucosamine within membranes (Vector, Burlingame, USA) to permit visualisation of membrane morphology.

To prevent photo-bleaching, myocytes were mounted in vectorshield (Vector, USA) and all slides were stored in the dark at 4 °C prior to examination by laser scanning confocal microscopy (Zeiss, Hertfordshire, UK). All fluorescent images were individually collected for each optical slice, using the same microscope and settings for the lasers and detector, from superimposing 6–10 optical slices taken at ≤1 μm intervals at each wavelength. Images of each intercalated disc were analysed by measuring their axes and area using the confocal software (LSM, Zeiss). Analysis of images such as density of label were performed using ImageJ v2.11x (NIH, USA).

### 2.4 Western blot

Tissue was snap-frozen and processed for analysis. Samples (50 μg total protein/lane) were separated by electrophoresis under reducing conditions by 10% SDS-PAGE, followed by transfer to a nitrocellulose membrane (Jones et al., 2004). The membrane was immersed and agitated in superblock TBS buffer (Pierce biotechnology, UK) for 40 min, then rinsed in TBS. Membranes were incubated overnight with appropriate primary antibodies (see Table 1) at 4 °C. The membrane was washed in Tween20 TBS, incubated for one hour with anti-rabbit or anti-mouse IgG conjugated to horseradish peroxide (HRP) (Dako, Germany), and washed in TBS. The membrane bound protein-antibody complex was detected using SuperSignal West Pico chemiluminescent substrate and CL-XPosure film (Pierce, Thermo Scientific, UK), bands were analysed using ImageJ. Total endogenous Cx43 protein was calculated using a commercial control of known total Cx43 protein content (Chemicon, UK), then expressed per μg total protein (μg total Cx43/μg total protein). In addition to the protein of interest, desmin protein levels were assessed to ensure equal protein loading of all samples determined by the uniform-density of desmin bands at the molecular weight of 53 kDa.

### 2.5 Direct inhibition and activation of JNK in vitro

Single cardiac myocytes isolated from adult animals (*n* = 5) were placed on cover-slips and either pre-exposed for 30 min to the JNK inhibitor SP600125 (30 μM anthra[1,9-cd]pyrazol-6(2H)-one in the carrier ≤0.1% DMSO) or the JNK activator anisomycin at 10 ug/ml for 90 min (Bennett et al., 2001; Ogawa et al., 2004; Petrich et al., 2002). Following exposure myocytes were either frozen for western blot analysis or plated on polysine slides and immediately fixed using 4% para-formaldehyde for immunofluorescence. Myocytes were also exposed to 0.1% DMSO alone to confirm DMSO was a carrier only with no other effects.

### 2.6 Statistical analysis

Data are expressed as means ± SEM and statistical differences assessed by ANOVA with Holm-Sidak post-hoc comparisons or Student’s *t*-test as appropriate. Differences were regarded as significant if *p* < 0.05. Correlations were determined using linear regression or Pearson correlation.

## 3 Results

### 3.1 Analysis of total endogenous connexin in right atrial muscle

**Fig. 1:**
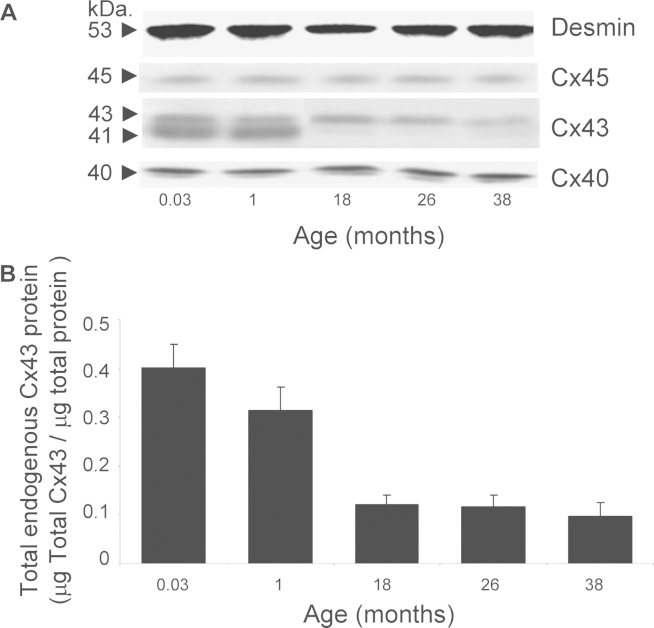
Endogenous connexin expression in right atrial muscle. (A) Illustrative membranes from western blotting are shown for the connexin proteins: Cx45 (single band at 45 kDa), Cx43 doublet (band at 43 kDa is phosphorylated Cx43, the band at 41 kDa is non-phosphorylated Cx43) and Cx40 (single band at 40 kDa). Equal protein-loading was confirmed by desmin labelling (53 kDa). (B) Total endogenous Cx43 protein (μg) per total protein (μg) significantly declined with ageing (*n* = 5; ANOVA *p* < 0.001).

Right atrial muscle from guinea pigs of 0.03 month (1 day) to 38 months of age (*n* = 5 per age group) was analysed by western blot for changes in total endogenous protein; Cx40 (bands at 40 kDa), Cx43 (doublet bands consisting of two isoforms; phosphorylated at 43 kDa and non-phosphorylated at 41 kDa) and Cx45 (bands at 45 kDa) (Fig. 1A). Equal protein loading was confirmed by uniform density bands of desmin protein at 53 kDa (Fig. 1A). The example membrane image illustrates the decrease in total endogenous Cx43 protein expression with increasing animal age. In contrast, Cx40 and Cx45 expression remain the same across the age range (Fig. 1A). Progressive ageing significantly reduced total endogenous Cx43 protein in the right atria to only 24 ± 2% of that identified in the neonate by 38 months of age. Total Cx43 protein declined from 0.40 ± 0.05 μg/μg at 0.03 months to 0.31 ± 0.05 μg/μg at 1 month, significantly dropping to 0.12 ± 0.02 μg/μg at 18 months and then was further reduced to 0.09 ± 0.03 μg/μg at 38 months of age (Fig. 1B; ANOVA, *p* < 0.001).

### 3.2 Dimensions of the intercalated disc increase with age

**Fig. 2:**
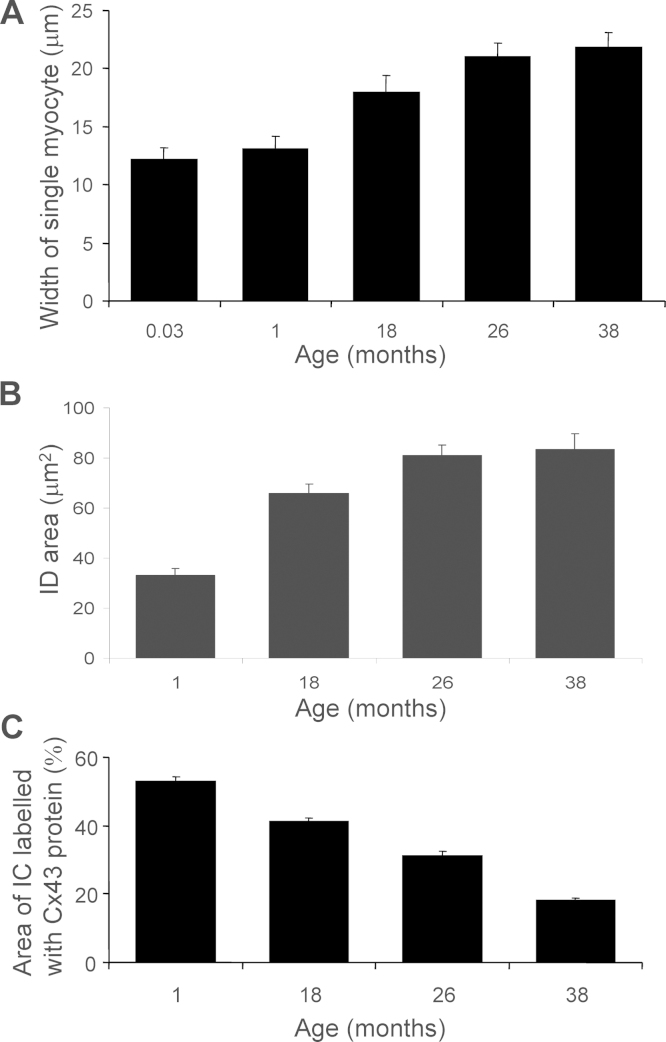
Dimensions of the intercalated disc. Myocyte width (A) and intercalated disc area (B) significantly increased between age groups (*n* = 30; ANOVA *p* < 0.001). Intensity of total endogenous Cx43 protein from each intercalated disc expressed as a percentage of the total disc area (C) was found to decline with age (*n* = 50; ANOVA *p* < 0.0001; linear regression, *y* = −0.94x + 55.4).

The cell width, intercalated disc major and minor axes measurements, and intercalated disc area was recorded from single isolated myocytes, obtained from the right atrial muscle for each age group (*n* = 30 myocytes per group). The width of myocytes significantly increased with ageing from 12.19 ± 0.9 μm at 0.03 month to 21.81 ± 1.3 μm at 38 months (Fig. 2A; ANOVA, *p* < 0.001). This cellular hypertrophy appears to be a normal developmental response, with no significant alteration in myocyte width occurring between 18 and 38 months of age (17.92 ± 1.5 μm at 18 months compared with 21.81 ± 1.3 μm at 38 months). The lack of significant additional hypertrophy with advancing age has previously been demonstrated for this model, as the heart weight does not change between the 18 month to 38 month old animal and the heart-to-body weight ratio remains constant across all ages studied (Jones et al., 2004).

The area of intercalated disc labelled positive for total endogenous Cx43 protein was defined by the area within the perimeter Cx43 signal. When the intercalated disc was examined it was found that both axes significantly increased; the major axis from 7.79 ± 0.36 μm at 1 month to 12.28 ± 0.47 μm at 38 months and the minor axis from 5.23 ± 0.25 μm at 1 month to 8.79 ± 0.35 μm at 38 months (Table 2; ANOVA *p* < 0.001). The average individual intercalated disc area significantly increased over the animal's lifespan from 33.0 ± 2.8 μm^2^ at 1 month to 83.0 ± 6.3 μm^2^ at 38 months significantly correlating with progressive ageing (Fig. 2B; *n* = 30 per age group; ANOVA *p* < 0.001; linear regression, *y* = 1.423x + 36.2; *R*^2^ = 0.91).

### 3.3 Intercalated disc total endogenous Cx43 labelling reduces with age

The area of intercalated disc labelled positive for total endogenous Cx43 protein, measured as the total intensity of Cx43 immunofluorescent signal, was expressed as a percentage of the total area of the intercalated disc (as defined by the area within the perimeter Cx43 signal). At 1 month of age 52.8 ± 2% of the intercalated disc labelled positive for Cx43 protein decreasing to 41.3 ± 1% at 18 months and 32.0 ± 1% at 26 months. By 38 months of age, the area showing expression of Cx43 protein was 18.0 ± 1 % of the total disc area. Overall, across the age range studied total Cx43 at the atrial myocyte intercalated disc significantly declined to 34% of the original content, a process which significantly correlated with age (Fig. 2C; *n* = 50; ANOVA *p* < 0.001; linear regression, *y* = −0.94x + 55.4; *R*^2^ = 0.96).

### 3.4 Remodelling of total Cx43 at the intercalated disc occurs with age

**Fig. 3:**
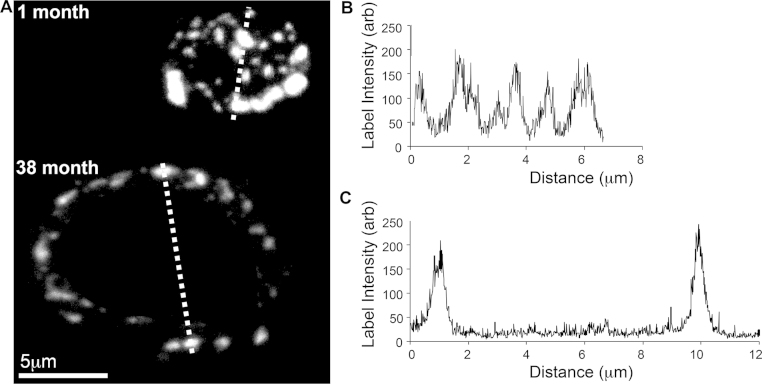
Example projection confocal images of intercalated discs with Cx43 labelled from animals of 1 month and 38 months of age (A). For the position indicated by the dashed line that bisects each disc image (A) an example intensity profile of Cx43 distribution across the intercalated disc from animals at (B) 1 month and (C) 38 months of age is shown.

**Fig. 4:**
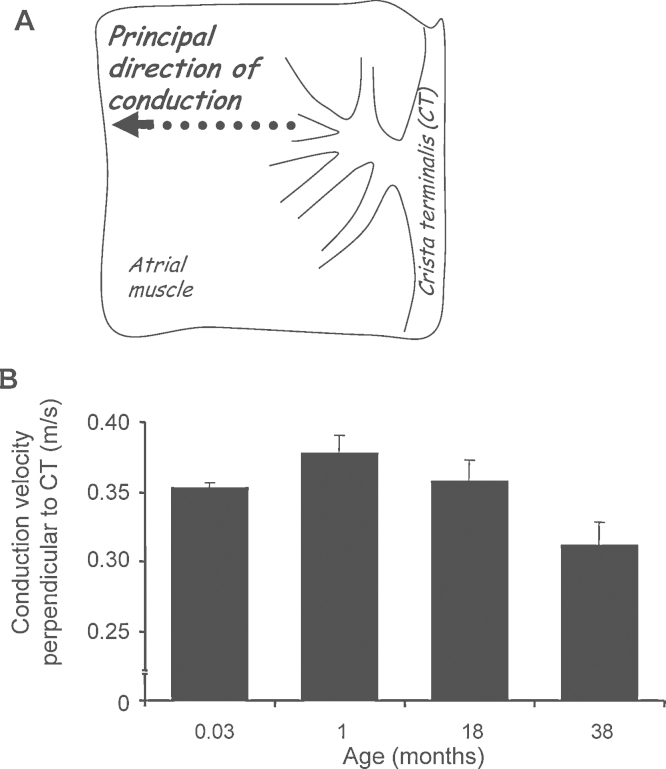
Conduction velocity in right atrial muscle of animals from 1 day to 38 months of age. Endocardial action potential conduction velocity was measured perpendicular to the crista terminalis (CT) as denoted by the dotted arrow in panel A. The conduction velocity was increased at 1 month compared with the neonate but conduction declined over the remaining ages until 38 months (B) (*n* = 5 per age group; ANOVA *p* < 0.01; linear regression, *y* = −0.0018x + 0.38).

The area of the intercalated disc increases with age, but in contrast the amount of total endogenous Cx43 protein expressed at the disc reduced with increased age, therefore spatial re-modelling of the distribution of the remaining Cx43 protein occurs. Intensity profiles of fluorescently-labelled endogenous Cx43 across each intercalated disc were measured. In Fig. 3A, an illustrative dashed line bisects the representative images of an intercalated disc for atrial myocytes obtained from animals at the ages 1 month and 38 months. The intensity profile of the representative intercalated disc from the 1 month old animal illustrates that many Cx43 ‘hot spots’ were present across the entire disc, demonstrated by the multiple peak profile (repeated observation *n* = 40 intercalated discs for 1 month; Fig. 2B). The intensity profile of the representative intercalated disc from the 38 month animal showed that labelled Cx43 protein was present at the outer rim only (repeated observation *n* = 40 intercalated discs for 38 month; Fig. 1C). Therefore, with increasing age the spatial distribution of Cx43 protein changes from consistent expression across the disc to being present at the outer rim only. The functional consequences of this remodelling were ascertained as shown in Fig. 4.

In contrast to this microscopic remodelling of the Cx43 expression the macroscopic apparent distribution of Cx43 remained unchanged with Cx43 remaining located at the cell ends with no signs of lateralisation.

### 3.5 Increased age slows the conduction velocity in right atrial muscle

For consistent assessment of the endocardial conduction velocity of the action potential propagation in the dominant direction of spread was measured, perpendicular to the crista terminalis (CT) (Fig. 4A). Between animals aged 1 day–1 month the conduction velocity increased from 0.35 ± 0.004 m/s (*n* = 5) to 0.38 ± 0.012 m/s (*n* = 4). However, from 1 month of age onwards over the remaining lifespan the conduction velocity significantly reduced to 0.31 ± 0.017 m/s at 38 months of age (*n* = 6) (Fig. 4B; ANOVA *p* < 0.01). This atrial muscle conduction velocity significantly negatively correlated with age (linear regression, *y* = −0.0018x + 0.38; *R*^2^ = 0.97).

### 3.6 Phosphorylation status changes with age

**Fig. 5:**
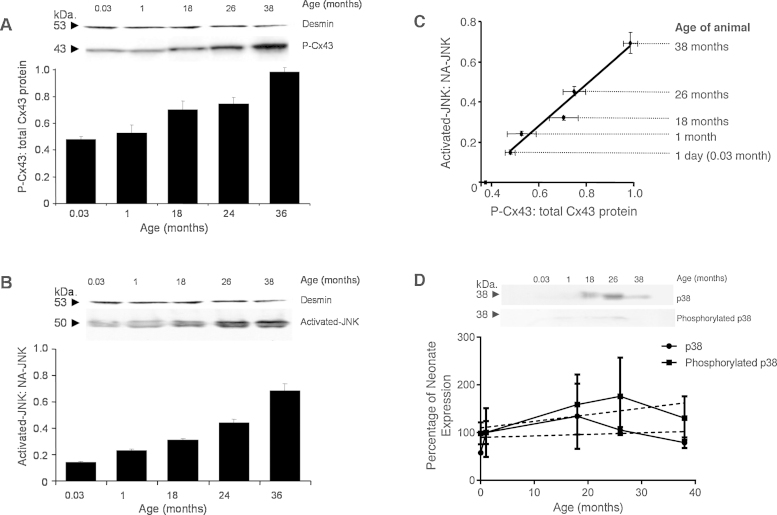
The representative membrane (A) illustrates the changes in phosphorylated Cx43 (P-Cx43) with age, expressed as the ratio of P-Cx43 to total Cx43 protein. (B) Illustrates the changes in activated-JNK with age, expressed as a ratio of activated JNK to non-activated JNK (NA-JNK). (A–B) Desmin content is shown to verify equal protein loading. (C) Shows the correlation between the ratio of proteins activated-JNK:NA-JNK and P-Cx43:total Cx43 across the age range (*n* = 5 per age group; ANOVA *p* < 0.01; linear regression *y* = 1.0305X–0.3484 = 0.96). (D) p38 (upper blot) and phosphorylated p38 (lower blot) at each age with average data shown below (mean ± SD). The dashed lines show linear regressions to the data. There is no significant correlation between p38 or phosphorylated p38, and age (*R*^2^ = 0.03 and 0.42, respectively, by Pearson correlation).

Example membranes (Fig. 5A and B) illustrates how the specific isoforms of the proteins, phosphorylated Cx43 (P-Cx43) and activated-JNK (known also as phosphorylated JNK) increase with progressive ageing. For each sample of right atrial muscle examined, the level of P-Cx43 protein was expressed as a ratio of the total Cx43 protein. The ratio of P-Cx43:total Cx43 significantly increased from 0.48 ± 0.02 at 0.03 month to 0.99 ± 0.03 in the atria from the 38 month animals (Fig. 5A; *n* = 5; ANOVA *p* < 0.001; *y* = 0.0129x + 0.486; *R*^2^ = 0.97). Each sample was also assessed for levels of activated-JNK expressed as a ratio to the non-activated JNK (NA-JNK) protein (the non-phosphorylated isoform). The ratio of activated-JNK:NA-JNK significantly increased from 0.14 ± 0.01 at 0.03 month to 0.68 ± 0.05 in the atria of the 38 month animals (Fig. 5B; *n* = 5; ANOVA *p* < 0.0001; linear regression, *y* = 0.0131x + 0.1544; *R*^2^ = 0.92).

Across the examined age range the levels of phosphorylated isoforms for both proteins, Cx43 and JNK, increased. A correlation between the increase in the level of phosphorylated P-Cx43 and the increase in activated-JNK within the right atrial muscle was identified (Fig. 5C; *n* = 5 animal per age, per protein; linear regression, *y* = 1.0305x – 0.3484; *R*^2^ = 0.96). Right atrial tissue from the old animal contained almost entirely phosphorylated Cx43 protein (98.6 ± 3 P-Cx43:total Cx43).

In contrast to the age-correlated increase in activated JNK another key stress-related kinase-p38 and phosphorylated p38 did not change significantly between young and old, or senescent animals, and failed to show a significant correlation with age (Fig. 5D) suggesting the increase in JNK is not just a generic increase in stress-activated MAP kinases with age.

### 3.7 JNK manipulation directly associates with Cx43 changes

**Fig. 6:**
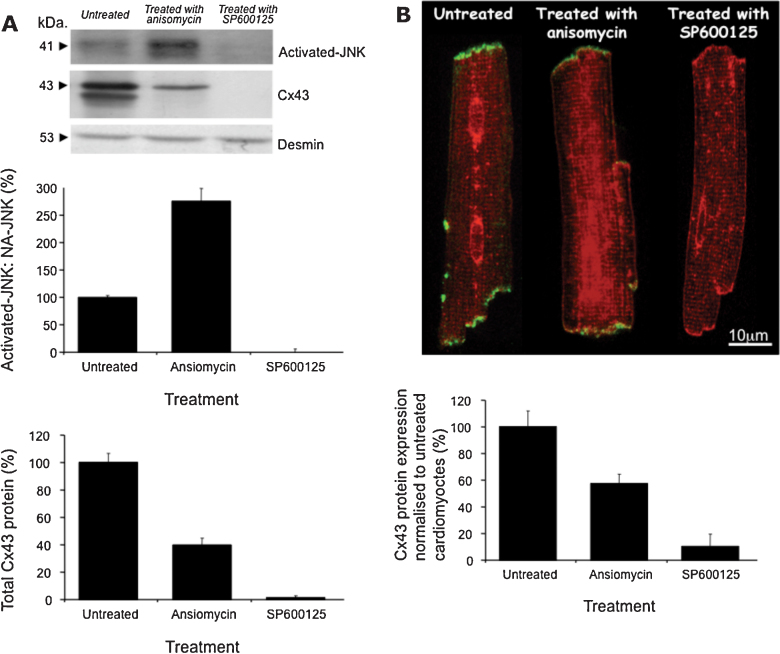
Isolated single cardiac myocytes were either treated with vehicle only (DMSO, control), exposed to the pharmacological agent SP600125 (JNK inhibitor) or anisomycin (an activator of JNK) an example membrane from western blot (A) illustrates the associated changes in Cx43 and JNK (desmin levels verify equal protein loading). Exposure to anisomycin increased activated-JNK (expressed as a ratio of activated-JNK to non-activated JNK) but SP600125 eliminated all trace of activated-JNK. Cx43 protein expression significantly declines in both the presence of anisomycin and SP600125 in comparison with the untreated myocytes (A) (*n* = 10; ANOVA, *p* < 0.01). The ‘untreated’ myocytes exposed to DMSO alone show Cx43 at the intercalated discs (B) (*n *= 20 per group). Myocytes exposed to anisomycin had significantly less Cx43 at the intercalated discs and SP600125 further reduced Cx43 labelling to visually undetectable levels (*n* = 20 per group, ‡*p* < 0.0001). The red labelling is from wheat germ agglutinin included as a membrane marker.

Finally we studied the controversial regulatory link between JNK and Cx43 using single cardiac myocytes isolated from healthy adult animals, exposed to either SP600125, a specific JNK inhibitor, or anisomycin, a specific activator of JNK, compared with ‘untreated’ cells in the carrier alone (Fig. 6). Western blot confirmed exposure to anisomycin increased activated-JNK to 273 ± 18%; however, SP600125 completely removed all detectable trace of activated-JNK protein (Fig. 6A; *n* = 10; ANOVA, *p* < 0.01). Total Cx43 protein levels declined in the presence of anisomycin to 41 ± 4% and SP600125 to 2 ± 1%.

WGA immunolabelling showed all examined myocytes had maintained their structure. Co-localisation of Cx43 protein and WGA, demonstrated that ‘untreated’ myocytes possessed Cx43 protein at the intercalated discs (Fig. 6B). Exposure to anisomycin significantly reduced Cx43 protein at the discs to 57 ± 7% of untreated myocytes, compared with SP600125 which instigated a comprehensive reduction to 11 ± 9% of that identified in untreated myocytes (Fig. 6B; *n* = 20 per group; ANOVA, *p* < 0.01).

## 4 Discussion

With progressive ageing there is an incrementing risk of atrial arrhythmias to the extent that age is considered one of the main risk factors for atrial fibrillation (Siddiqi and Dickfeld, 2011). This incrementing risk is associated with atrial conduction disturbances and disruption of the normal pacemaking of the heart (Jones et al., 2004). Conduction through the myocardium is dependent on tissue architecture, action potential shape, size of cells and coupling conductance between cells. In our previous work using this guinea-pig model from Massons-trichrome staining we failed to identify significant fibrosis/reorganisation of the atria with age in this animal model (Jones et al., 2004) (although this is certainly a key factor in many associated pathologies and appears to rapidly occur consequent to atrial fibrillation). Equally the modest hypertrophy of cells observed with age would be expected to increase conductance rather than lead to the decrease documented in this study. This leaves action potential changes and changes in connexins as the main potential problems that predispose to conduction problems in the aged atria. Age is associated with increased atrial action potential duration in several studies (for review see Dun and Boyden, 2009) but no differences in the upstroke of the action potential, which is key for determining propagation, have been reported and studies on canine atrial myocytes failed to find an age-associated change in sodium current (Baba et al., 2006).

This leaves connexins and the associated inter-cellular conduction as the likely key parameter responsible for determining changes in tissue conductivity in the atria with advancing age. Previous reports have shown that the conduction velocity across the right atria, although chiefly dependent on Cx43 expression, also decreases if more Cx40 protein is expressed relative to Cx43 protein expression as a consequence of lower channel conductance (Kanagaratnam et al., 2002). Analysis of protein expression with age by western blot determined that expression of Cx40 and Cx45 proteins did not alter with age; however, a significant progressive decrease of Cx43 protein expression levels occurred with ageing. This decline in Cx43 expression was predicted to lead to diminished electrical conductivity across the tissue. The associated increased resistance to electrical communication, decreased action potential conduction velocity and associated increased cardiac conduction times are factors purported to contribute to the generation of arrhythmias (Kanagaratnam et al., 2002; Kistler et al., 2004).

At birth the immunoreactive Cx43 signal is uniformly distributed around the myocyte perimeter but by 1 month, development of the heart is associated with gap junctional remodelling and Cx43 has relocated to the intercalated discs (Spach et al., 2000). Despite our observation that Cx43 expression declined significantly from 1 day to 1 month, the conduction velocity of the action potential in the direction perpendicular to the crista terminal slightly increased from 0.35 ± 0.004 m/s at 1 day to 0.38 ± 0.012 m/s at 1 month of age. The spatial remodelling of the Cx43 protein from the cellular perimeter to the intercalated disc and ensuing improved gap junctional alignment remodelling appears to produce an anisotropic effect that accompanies an increase in the speed of action potential conduction. Interestingly in the condition ‘Tetralogy of fallot’ this remodelling of Cx43 protein fails to occur, and this failure has been associated with an increased risk of arrhythmias highlighting how the distribution of Cx43 is an important factor in determining susceptibility to arrhythmias (Kołcz et al., 2005).

Stress-activated MAP kinases (SAPKs) are a subset of the MAP kinase family (MAPKs), including JNK and p38. These kinases respond to a variety of stresses including physical, chemical, metabolic and biological. Conditions that induce acute stress *in vivo*; for example, ischemic episodes, myocardial infarction and end-stage heart failure, are associated with increased levels of activated-JNK and loss of Cx43 proteins (Luke and Saffitz, 1991; Dupont, 2001). Exposure of cultured single cardiac myocytes to acute stress has been shown to stimulate increased activated-JNK, followed by down-regulation of Cx43 mRNA from 60 min onwards (Petrich et al., 2004). Post-stress Cx43 protein levels were found to be 60% of that in untreated myocytes. The potential *in vivo* consequence of this Cx43 loss is uncoupling of the functional myocardium from injured tissue, which is thought to be protective, electrically insulating the neighbouring myocytes and preventing extension of the infarct area (Luke and Saffitz, 1991). Whilst our data is not from acutely stressed hearts, it is acknowledged that even apparently healthy ageing can be a chronic stressor associating with induction of stress signalling pathways (Powers et al., 2004). This induction with ageing is seen in our finding that levels of activated-JNK increase with age correlating directly with reduced total Cx43. The only previous study to consider changes in JNK activation *in vivo* with age used a comparison between young and middle-aged rabbits and identified a 120% increase in JNK activation in the left atrium (Yan et al., 2013). In contrast our study across the lifespan has shown a more than 4 fold increase in the right atria associating directly with progressive ageing. This is not a simple global increase in stress kinases since p38 failed to show the same response.

Evidence of a direct link between JNK and Cx43 regulation in the heart comes from transgenic mice with cardiac-specific *in vivo* activation of JNK. These mice exhibit a phenotype of sudden and premature death, attributed to down-regulation of Cx43 expression, associated loss of gap junctions and slowed conduction (Petrich et al., 2004). These findings are closely paralleled by observations from mice with a cardiac-restricted knockout of Cx43, who at 45 days old possessed ∼20% Cx43 protein compared with their control littermates and a significantly slowed conduction velocity associating with an increase in their susceptibility to induced arrhythmias (Danik et al., 2004). Similar observations have also now been made with moderate ageing and induction of JNK in the rabbit heart where ageing from a juvenile state to middle age as well as anisomycin treatment both resulted in activation of JNK and a reduction in Cx43 expression (Yan et al., 2013).

Analysis of total endogenous Cx43 protein expression in our ‘ageing’ animal model found progressive ageing reduced the Cx43 protein expressed in the right atria to 25% of the original content by 38 months correlating with a significant increase in activated-JNK and a reduction in action potential conduction velocity in the right atrial muscle. A correlation previously observed within the sinoatrial node (Jones et al., 2004). Elderly patients suffering arrhythmic activity have previously been observed to possess slowed conduction of the action potential associated with reduced expression of Cx43 protein within the right atrium (Kistler et al., 2004; Kostin et al., 2002), a finding mirrored in our animal model. Thus similarities can be drawn between the old aged human heart, our aged animal model and the previously discussed transgenic mouse models: suggesting that electrical uncoupling is a result of the diminished Cx43 expression with JNK playing a mechanistic role in the formation of an arrhythmic substrate increasing the risk of sudden death.

Furthermore we investigated the controversial relationship between JNK and Cx43 expression. Anisomycin was used as an activator of JNK (Ogawa et al., 2004; Petrich et al., 2002), which in our hands also lead to increased phosphorylation of Cx43. The anthrapyrazlone inhibitor of JNK, SP600125, has been previously used to inhibit JNK pathways without affecting other mitogen-activated protein kinase (MAPK) pathways such as p38 MAPK or extracellular signal-regulated pathways (ERK) (Bennett et al., 2001). In our study cardiac myocytes were treated with SP600125 and this resulted in the complete abolishment of Cx43 protein expression, an event that did not occur in the untreated cells. This directly demonstrates the link between JNK signalling and the regulation of Cx43 expression, but does highlight a complex relationship whereby activation can cause phosphorylation and chronic down-regulation of Cx43, similarly complete inhibition causes a complete block of Cx43 expression. Further experiments are required to fully understand the interactions of this linked system.

Our data demonstrate that during healthy *in vivo* progressive ageing activated-JNK accumulates as total endogenous Cx43 protein declines and phosphorylation of Cx43 protein increases in the right atria. De-phosphorylation of Cx43 is known to lead to reduced conductance in ischemia with potential negative and beneficial consequences (Beardslee et al., 2000). Phosphorylation of Cx43 though is also known to increase Cx43 degradation (Lampe and Lau, 2000; Popolo et al., 2013). This apparent dichotomy plays out in the ageing heart in a manner whereby degradation increasingly predominates leading to depletion of Cx43. Counter to this the increasingly high proportion of phosphorylated Cx43 found in the ageing heart could therefore be considered as maximising conductance from the decreasing number of gap junctions expressed, a change which ultimately fails to maintain homeostasis due to the elevated degradation. Future work will be able to evaluate further the changes in protein turn-over with ageing and the role the activation of JNK plays in altering this. On the current data though it would appear the elevating activation of JNK with age is serving to drive the atria into a pro-arrhythmic state by ultimately inducing loss of Cx43 accompanying functional remodelling and reduced conduction.

## 5 Conclusions

We have provided novel evidence that JNK signalling is a mediator of gap junctional remodelling during progressive ageing of the right atria by a mechanism that could account for the observed dynamic changes in Cx43 protein expression. JNK activation can explain changes in conduction and associated connexin remodelling in the aged heart, providing an important link between the progressing chronic stress of ageing and increasing susceptibility to arrhythmias. Identification of the role of JNK in the aged heart requires further investigation to determine if it is a therapeutic target worth pursuing in elderly patients at risk from atrial arrhythmias. With an appropriately directed cardiac-specific pharmacological tool, the attenuation of activated-JNK could offset the age-dependent effects. Although not pharmacological, one intervention already known to attenuate the JNK stress-induced cell signalling mechanism providing protection to young and old hearts against stress-related damage is exercise (Boluyt et al., 2003; Kumar and Tatu, 2003; Powers et al., 2004). Thus, our data provides evidence how *in vivo* ageing manipulates the expression of Cx43 protein in the heart by the JNK pathway resulting in slowed cardiac action potential conduction and an increased risk of arrhythmias in the ageing population.

## Disclosure

There are no conflict of interest to disclose.

## Author contribution

Dr. Jones conducted the experiments and analysed the data, as well as drafting the manuscript.

Dr. Lancaster conducted the electrophysiological experiments and analysed the electrophysiological data as well as editing the manuscript.

## Figures and Tables

**Table 1:** The antibodies used in this study with concentrations used and their source companies.

Antibody	Source company	Host	Immunofluorescence	W-blot
			μg/ml	μg/ml
Cx40	Chemicon, Hampshire, UK	Rabbit	20	10
Cx45	Chemicon, Hampshire, UK	Mouse	20	10
Total endogenous Cx43	Cell signalling Tech., Hertfordshire, UK	Rabbit	1	0.1
Phosphorylated Cx43	Cell signalling Tech., Hertfordshire, UK	Rabbit	–	1
Non-phosphorylated JNK	Cell signalling Tech., Hertfordshire, UK	Rabbit	–	1
Phosphorylated JNK	Cell signalling Tech., Hertfordshire, UK	Rabbit	–	1
Desmin	Dako, Denmark	Mouse	–	6

**Table 2:** The size of the intercalated disc with age.

Age of animal	Major axis of the intercalated disc	Minor axis of the intercalated disc
(months)	(μm)	(μm)
1	7.79 ± 0.36	5.23 ± 0.25
18	10.56 ± 0.38	7.77 ± 0.27
26	12.10 ± 0.42	8.43 ± 0.19
38	12.28 ± 0.47	8.79 ± 0.35

The maximal distance across the intercalated discs was recorded as the major axis, and the minimal distance recorded as the minor axis (*n* = 30 for each age group; ANOVA *p* < 0.001).
